# Causal Mediation of Stress Reduction in Family Dementia Caregivers: A Focus on Mindfulness

**DOI:** 10.1093/geroni/igab046.678

**Published:** 2021-12-17

**Authors:** Liliana Ramirez Gomez, Felipe Jain

**Affiliations:** Massachusetts General Hospital, Harvard Medical School, Boston, Massachusetts, United States

## Abstract

Although mindfulness therapies have demonstrated benefits for reducing stress and improving psychological symptoms in family dementia caregivers, the mechanisms underlying these salutary effects are unknown. We report a causal mediation pathway to improvement of stress symptoms in family dementia caregivers with Mentalizing Imagery Therapy (MIT), which employs mindfulness and guided imagery tools to reduce stress and improve understanding of self and others. In a randomized controlled trial of short-term 4-week MIT (N=24) versus a psychosocial support group (N=22), MIT demonstrated superior benefit for reducing perceived stress (p=.006). Increased trait mindfulness was a causal mediator of this effect (p=.02). Neuroimaging pre and post intervention found that increased mindfulness was associated with strengthened connectivity of the dorsolateral prefrontal cortex with an emotion regulation network (p<.001). The results are discussed in light of theories of cognitive control and may inform the design of future studies aimed at reducing family caregiver stress.

